# Personalized Antihypertensive Treatment Optimization With Smartphone‐Enabled Remote Precision Dosing of Amlodipine During the COVID‐19 Pandemic (PERSONAL‐CovidBP Trial)

**DOI:** 10.1161/JAHA.123.030749

**Published:** 2024-02-07

**Authors:** David J. Collier, Mike Taylor, Thomas Godec, Julian Shiel, Rebecca James, Yasmin Chowdury, Patrizia Ebano, Vivienne Monk, Mital Patel, Jane Pheby, Ruby Pheby, Amanda Foubister, Clovel David, Manish Saxena, Leanne Richardson, James Siddle, Gregor Timlin, Paul Goldsmith, Nicholas Deeming, Neil R. Poulter, Rhian Gabe, Richard J. McManus, Mark J. Caulfield

**Affiliations:** ^1^ William Harvey Research Institute Queen Mary University of London London UK; ^2^ Closed Loop Medicine London UK; ^3^ TrialsConnect London UK; ^4^ Imperial College Clinical Trials Unit, School of Public Health, Imperial College London London UK; ^5^ Wolfson Institute of Population Health Queen Mary University of London London UK; ^6^ Nuffield Department of Primary Care Health Sciences University of Oxford Oxford UK

**Keywords:** amlodipine, home blood pressure, hypertension, personalized dose titration, remote care, Hypertension, Primary Prevention, Clinical Studies

## Abstract

**Background:**

The objective of the PERSONAL‐CovidBP (Personalised Electronic Record Supported Optimisation When Alone for Patients With Hypertension: Pilot Study for Remote Medical Management of Hypertension During the COVID‐19 Pandemic) trial was to assess the efficacy and safety of smartphone‐enabled remote precision dosing of amlodipine to control blood pressure (BP) in participants with primary hypertension during the COVID‐19 pandemic.

**Methods and Results:**

This was an open‐label, remote, dose titration trial using daily home self‐monitoring of BP, drug dose, and side effects with linked smartphone app and telemonitoring. Participants aged ≥18 years with uncontrolled hypertension (5–7 day baseline mean ≥135 mm Hg systolic BP or ≥85 mm Hg diastolic BP) received personalized amlodipine dose titration using novel (1, 2, 3, 4, 6, 7, 8, 9 mg) and standard (5 and 10 mg) doses daily over 14 weeks. The primary outcome of the trial was mean change in systolic BP from baseline to end of treatment. A total of 205 participants were enrolled and mean BP fell from 142/87 (systolic BP/diastolic BP) to 131/81 mm Hg (a reduction of 11 (95% CI, 10–12)/7 (95% CI, 6–7) mm Hg, *P*<0.001). The majority of participants achieved BP control on novel doses (84%); of those participants, 35% were controlled by 1 mg daily. The majority (88%) controlled on novel doses had no peripheral edema. Adherence to BP recording and reported adherence to medication was 84% and 94%, respectively. Patient retention was 96% (196/205). Treatment was well tolerated with no withdrawals from adverse events.

**Conclusions:**

Personalized dose titration with amlodipine was safe, well tolerated, and efficacious in treating primary hypertension. The majority of participants achieved BP control on novel doses, and with personalization of dose there were no trial discontinuations due to drug intolerance. App‐assisted remote clinician dose titration may better balance BP control and adverse effects and help optimize long‐term care.

**Registration:**

URL: clinicaltrials.gov. Identifier: NCT04559074.

**Nonstandard Abbreviations and Acronyms**
AE: adverse event
EOT: end of treatment

**Clinical Perspective**

**What Is New?**

- This was the first trial to evaluate milligram‐by‐milligram personalized novel amlodipine dose titration for mild–moderate hypertension under a remote medical management protocol; novel doses of amlodipine were safe and effective in precision dosing for hypertension, up to the maximum licensed dose.
- This trial demonstrated the ability to remotely triage participants according to baseline blood pressure control, then adjust and personalize drug dose to optimize clinical outcome from information recorded by participants in a dedicated smartphone app and transmitted securely to a clinician.

**What Are the Clinical Implications?**

- Clinicians working remotely using personalized dose titration, assisted by a smartphone app, can achieve high patient adherence, regardless of age, and better balance blood pressure control and adverse effects to optimize long‐term care.

Hypertension is the leading preventable cause of morbidity and premature death worldwide.^1^ Globally, 1.4 billion people are estimated to have hypertension, which caused ≈10.7 million deaths in 2015 and is projected to affect >1.5 billion people globally by 2025.^2^, ^3^ In England, where 13.5 million people have hypertension, it causes >50% of all strokes and heart disease, accounting for 12% of general practitioner visits and over £2.1 billion of National Health Service costs.^4^ In the United States, it contributed to almost half a million deaths in 2018, which cost $131 billion.^5^ Hypertension is poorly controlled even when identified, and global estimates suggest only 13.8% of treated patients achieve blood pressure (BP) control.^1^ Management involves repeated visits and BP measurements in the clinic and, increasingly, at home. Clinician access to patient self‐monitored data, quality control, and processing of data are all challenging. Self‐monitoring combined with self‐titration of conventional antihypertensive drug doses has been shown to lower BP in people with hypertension^6^ but only the most tailored interventions have been effective.^7^

Among antihypertensive agents, calcium channel blockers, such as amlodipine, are first‐line recommended drugs in the United Kingdom for people >55 or Black persons of African‐Caribbean origin of any age. Amlodipine powerfully prevents cardiovascular events,^8^, ^9^ but ≈20% of patients started on amlodipine discontinue after a single prescription.^10^ Amlodipine dosing could be more effective or safer for more patients if increased dosing precision was available. The reduction in BP achieved with amlodipine is proportional to the dose taken but if a critical dose is exceeded, patients experience unacceptable side effects.^11^ The most commonly reported side effect hindering compliance with amlodipine is peripheral edema, which is strongly dependent on dose.^11^, ^12^

The usefulness of a therapy depends on a combination of effectiveness and patient adherence to the regimen. Poor patient adherence to prescribed treatment hampers chronic therapy for symptomless risk factors such as hypertension.^13^ Proper evaluation of antihypertensive therapy requires assessment of tolerability and effectiveness (BP control). Poor tolerability damages patient adherence and “Increasing the availability and affordability of these more precise measures of adherence represent a future opportunity to realize more of the proven benefits of evidence‐based medications…, it is important that health care providers focus their attention on how to do better with the drugs they have.”^14^

Hence amlodipine was chosen for precision dosing for hypertension using novel doses and personalization of dose, for which a digital solution was developed for recording wanted and unwanted effects. This tailored digital solution was repurposed for the COVID‐19 pandemic using a decentralized clinical trial platform to allow remote recruitment, remote assessment of BP control, and for those found to have poor control of BP, a personalized amlodipine dose titration protocol incorporating novel doses. The key advantage of amlodipine in the remote care setting during lockdown was that it could be introduced more simply than most other BP‐lowering drugs, in part because blood tests to check renal function are not required (which are needed before safely starting an angiotensin‐converting enzyme inhibitor, an angiotensin II receptor antagonist, or diuretic drugs). As the titration course with amlodipine could last up to 14 weeks, the use of a placebo was considered against patients' interests. The resultant PERSONAL‐CovidBP (Personalised Electronic Record Supported Optimisation When Alone for Patients With Hypertension: Pilot Study for Remote Medical Management of Hypertension During the COVID‐19 Pandemic) trial was designed to evaluate whether a drug plus digital intervention (comprising self‐monitoring of BP and side effects) and clinician‐led drug dose changes would result in lower systolic BP (SBP) in people with poorly controlled hypertension.

## Methods

Data that support the findings of this trial are available from the corresponding author upon reasonable request.

### Trial Design and Oversight

PERSONAL‐CovidBP was an open‐label, remote care, personalized, nonrandomized, community‐based trial, which recruited eligible participants from October 2020 to July 2021.

This trial was reviewed and approved by Dulwich Research Ethics Committee, the Health Research Authority, and the Medicines and Healthcare Products Regulatory Agency, which provided a favorable opinion to conduct this trial in the United Kingdom. Written informed consent was obtained from all participants. The trial was overseen by a trial steering committee.

The trial was conducted in accordance with ethical principles consistent with the Declaration of Helsinki, the International Council for Harmonization Good Clinical Practice guidelines, and all other relevant national and regional guidelines and regulations, including The Medicines for Human Use (Clinical Trials) Regulations 2004 (SI 2004/1031), and amendments.

All the authors have vouched for the adherence to the protocol, for the accuracy and completeness of data, and for the reporting of serious adverse events (AEs). The authors followed the Consolidated Standards of Reporting Trials (CONSORT) guidelines while preparing this article.^15^

**Figure 1:**
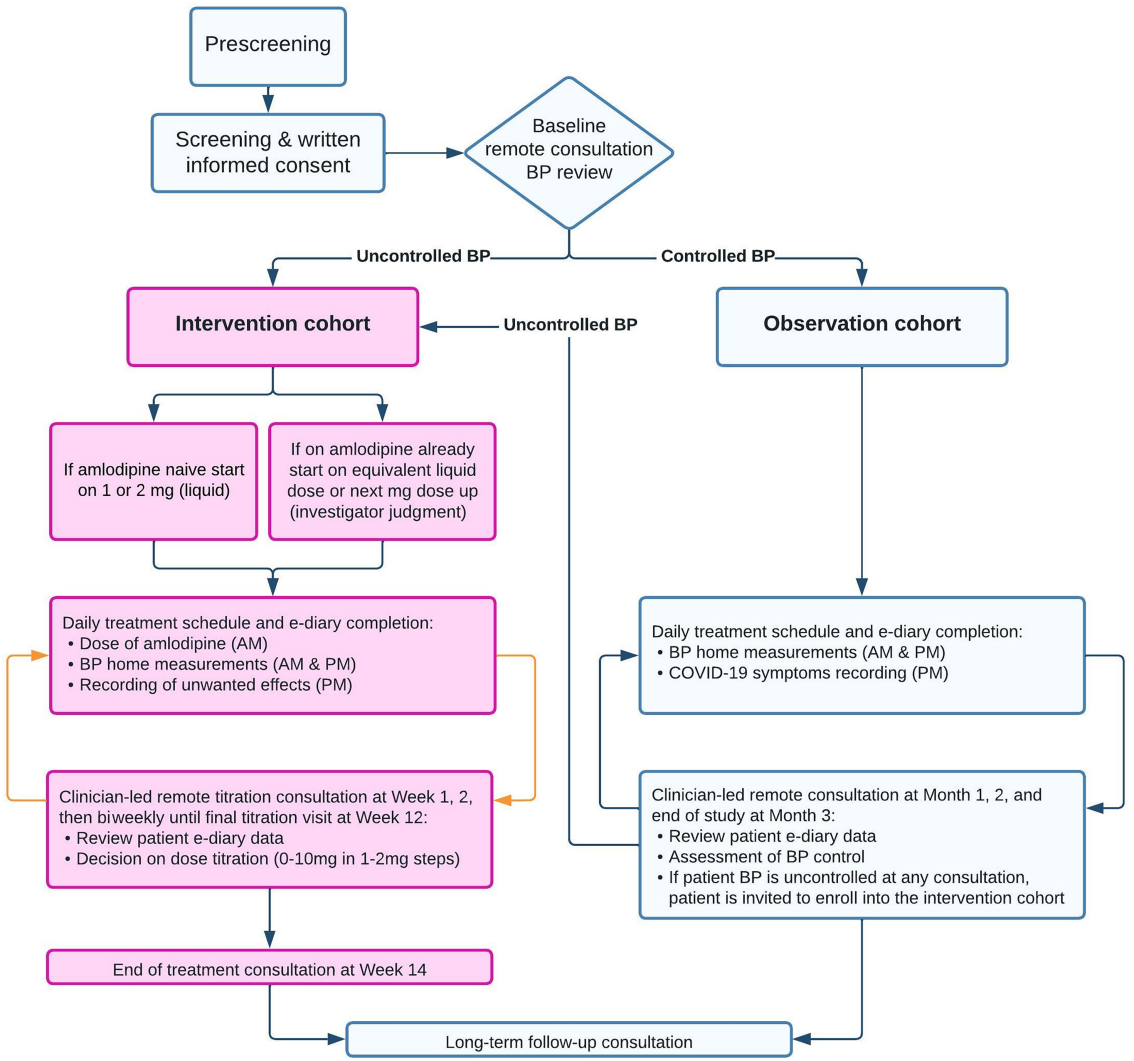
Trial protocol flow chart. BP indicates blood pressure.

The trial design is shown in Figure 1 and described next.

### Participants

Participants were aged 18 years and older with hypertension defined as either (1) participant account of a diagnosis of hypertension consistent with National Institute for Health and Care Excellence/British and Irish Hypertension Society criteria on either 24 hours ambulatory BP monitoring or repeated home measures of BP, ideally before treatment; or (2) current treatment with antihypertensive medication.

At prescreening, conducted remotely for the majority, participants gave verbal consent to sharing medical history and had to have hypertension as defined, which could include remote assessment of home recordings over 7 days before the screening visit. Main exclusions were already being on or above the maximum licensed dose of amlodipine (≥10 mg daily), current infection, or symptoms suggestive of COVID‐19 at the time of screening (rescreening when recovered was allowed), not having a suitable smartphone, known severe adverse reaction to amlodipine, and comorbidities incompatible with trial participation, including those that could result in a participant being unable to complete daily entries satisfactorily via their smartphone and also such comorbidities as determined by the principal investigator that could confound trial assessments. All other existing BP‐lowering drugs that participants were receiving were permitted to be continued, providing their doses could remain stable for the duration of the trial (allowing for unexpected changes for participant safety).

Participants who did not have a suitable home BP machine (upper arm cuff, validated design, and <4–5 years old)^16^ were sent a new Omron M3 Comfort home BP machine with a universal cuff. Training on the use of a BP monitor was provided during the screening consultation.

At screening, following completion of the written, or more usually e‐consent process (DocuSign), consenting participants downloaded the app onto their smartphone and began a baseline period of recording 3 BP measurements each morning and evening over 5 to 7 days. Home BP readings were manually entered into the app. A minimum of 5 days and 24 BP measurements were required to calculate baseline BP and to determine eligibility for intervention or observation cohort. Participants with a baseline mean of ≥135 mm Hg SBP or ≥85 mm Hg diastolic BP (DBP) were enrolled to the intervention cohort and received personalized dosing with amlodipine over 14 weeks. Amlodipine dose options included novel (1, 2, 3, 4, 6, 7, 8, and 9 mg) and standard daily doses (5 and 10 mg). The treatment approach allowed for adjustment of dose to zero when necessary to allow participant and clinician to assess causality of effects; this allowed continuation in the trial and for the reintroduction of amlodipine where possible and also allowed participants to be open about unwillingness to restart amlodipine while remaining adherent to the therapeutic approach.

Participants with controlled BP (mean SBP <135 and DBP <85 mm Hg) during the 5‐ to 7‐day baseline period were entered into an observation cohort. Participants in the observation cohort continued to use the app as downloaded at screening and continued to enter BP. Participants in the observation cohort were offered entry into the intervention part of the trial if their BP subsequently rose to ≥135 mm Hg SBP or ≥85 mm Hg DBP.

Intervention cohort participants downloaded a new version of the app optimized to capture unwanted effects of amlodipine, drug dose, and time taken as well as BP (3 measurements each morning and evening) and were provided with liquid amlodipine 1 mg/1 mL authorized as a prescription‐only medicine in the United Kingdom (Rosemont Pharmaceuticals) by courier under appropriate supply chain conditions. Product was stored and used in accordance with manufacturer's storage instructions and shelf life (closed bottle/open bottle) of 3 years/30 days. Amlodipine dose was measured using the oral syringe provided by the manufacturer and in accordance with the manufacturer's Patient Information Leaflet (graduated in 1 mg and 0.5 mg increments).

Suboptimal BP control was defined as mean SBP of 135 mm Hg or greater, or mean DBP of 85 mm Hg or greater during the run‐in period for entry into the intervention cohort. This was amended during the trial at the trial steering committee's suggestion from mean SBP 140 mm Hg or greater or DBP 90 mm Hg or greater to align it with British and Irish Hypertension Society/National Institute for Health and Care Excellence hypertension diagnostic criteria from self‐measured home BP.

### Trial Procedures

Trial consultations for participants were conducted remotely. All contact with the research trial team was held remotely via a teleconference using a mobile phone, laptop, personal computer, or telephone (in practice almost all contact was by telephone). Trial participants took part in teleconsultations at agreed‐upon times. Clinical members of the trial team—clinical scientists (n=3), research nurses (n=3), doctors (n=2)—contacted participants at least once every 2 weeks. Calls to participants in the observation arm were undertaken by nurses or clinical scientists. Doctors and nurses conducted the titration calls to participants in the intervention arm.

Intervention cohort participants had a remote consultation via tele‐call at least every 2 weeks while on treatment (from Baseline [Week 0] to Week 14). This involved the review of their BP and adjustment of medication dosage as indicated depending on all other smartphone app‐recorded data including side effects. Trial dose titration teleconsultations (and coinciding adherence checks) were scheduled twice weekly as pharmacokinetic‐pharmacodynamic studies show that it may take approximately 2 weeks before amlodipine exerts its full effect, which agrees with the clinical experience of the principal investigator. Adherence to monitoring routines was checked every 2 weeks by completeness of data entry and self‐reported outcomes by the participant. Adjustment of amlodipine dose for most participants was completed by the last titration visit (Week 12). A follow‐up call was made to ensure that participants in the intervention cohort were stable on posttrial treatment.

The observation cohort was not designed to serve as a direct comparator for the interventional cohort, as the 2 cohorts were inherently not comparable in terms of baseline BP (separation of cohorts was based on their baseline BP). Entrants to the observational cohort had adequately controlled BP and did not require the same frequency of review as those in the interventional cohort who had a dose titration consultation at least biweekly. The purpose of the observational cohort was to provide safety monitoring (3‐month) follow‐up with appropriate consultation frequency (monthly) and also to allow participants whose BP control became inadequate to be considered for entry to the interventional cohort (24 observational cohort participants transitioned to interventional cohort).

### Outcomes

The primary outcome was the mean change in SBP. This was measured daily at home from baseline to the end of treatment (EOT, Week 14) comparing 7‐day mean SBP at baseline (minimum of 24 values) to 7‐day SBP mean at EOT.

Secondary outcomes included: mean change in daily DBP (measured as previously described for SBP); proportion of participants reaching BP targets (either SBP <135 mm Hg and DBP <85 mm Hg, or drop in SBP ≥10 mm Hg, or drop in DBP ≥5 mm Hg) by EOT; treatment‐emergent AEs defined as AEs that were considered at least possibly related to trial treatment; reports of side effects using the app; patient‐reported outcome data including user experience; adherence to medication (% intervention participants reporting adherence to prescribed amlodipine dose on 80% or more days); adherence to BP monitoring routines (% participants making a BP record entry on 80% or more occasions, based on 2 occasions/day). The observation cohort was not designed to assess adherence to participants' background medication usage nor incidence of side effects given the large variation in number and drug class of antihypertensive background medications in use.

### Data Collection

After completion of a run‐in period of 5 to 7 days, participants' home BP (morning and evening), drug dose, and side effects were recorded daily via the app for a period of up to 14 weeks.

### Statistical Analysis

It was calculated that the enrollment of 200 participants would provide >90% power to detect a conservative change in SBP of 3.0 mm Hg from baseline to EOT with an SD of SBP change of 11 mm Hg, at the 5% significance level. Assumptions for SD were taken from the PATHWAY‐2 (Prevention and Treatment of Hypertension With Algorithm Based Therapy‐2) trial, where a 4.1 mm Hg change in SBP was observed from baseline in the placebo arm.^17^ This sample size allowed for >20% missing data from either loss to follow‐up or noncompliance while maintaining at least 90% power.

For the primary outcome of change in SBP from baseline to EOT in the intervention cohort, a participant's mean SBP was calculated at baseline and each scheduled visit up to EOT visit, as the mean of 7 days of SBP measurements preceding each visit, including all available BP entries for each participant during those time periods. First, all available BP values were used within a single routine to calculate a mean for that routine (ie, morning and evening each day were separate routines). Each BP routine would ask the patient for 3 BP measurements, with a fourth measurement prompted to be taken if the first 3 were substantially varied. A mean for a specific time point, for example, at baseline or EOT, was then calculated as the mean of all routine means over the 7 days (maximum of 14 routines) for each participant.

The primary end point was analyzed using a linear mixed effects model. The outcome in the model was SBP level and contained a fixed effect for visit, including baseline and each scheduled consultation up to EOT, and a random intercept component at the participant level to account for correlation between SBP measurements across visits within the same participant. The mean change in SBP between baseline visit and EOT visit was estimated from the model as the difference between SBP level at EOT and baseline, along with 95% CI and *P* value.

All 205 intervention participants who had BP measurements at least at 1 time point were included in the main intention‐to‐treat analysis. The primary end point was reanalyzed for a per protocol population consisting of intervention participants who had at least 24 BP measurements in the 7‐day window preceding EOT visit.

Subgroup analyses were performed for the primary outcome on subgroups of age group (<65, 65+ years), sex, race (Asian, Black, White, Other/Mixed), diabetes status, baseline antihypertensive medication (on no antihypertensive medication, on amlodipine [maximum 5 mg] alone, on amlodipine [maximum 5 mg] in combination with other antihypertensive[s]; or on other antihypertensive[s] not amlodipine).

All secondary end points were analyzed and presented as descriptive statistics, without statistical testing, including BP changes in the observation cohort.

### Patient and Public Involvement

Patient representatives contributed to several aspects of the trial, including approving fractional dosing, app design feedback in iterative user experience testing with Closed Loop Medicine, trial design and documentation of the grant bid submitted to Innovate UK, the choice of liquid amlodipine delivery (syringe), and the patient‐facing materials.^18^ A patient representative (N.D.) was an active member of the trial steering committee. Innovative forum theater techniques were employed with patients who had had past intolerance to amlodipine.^19^

## Results

**Figure 2:**
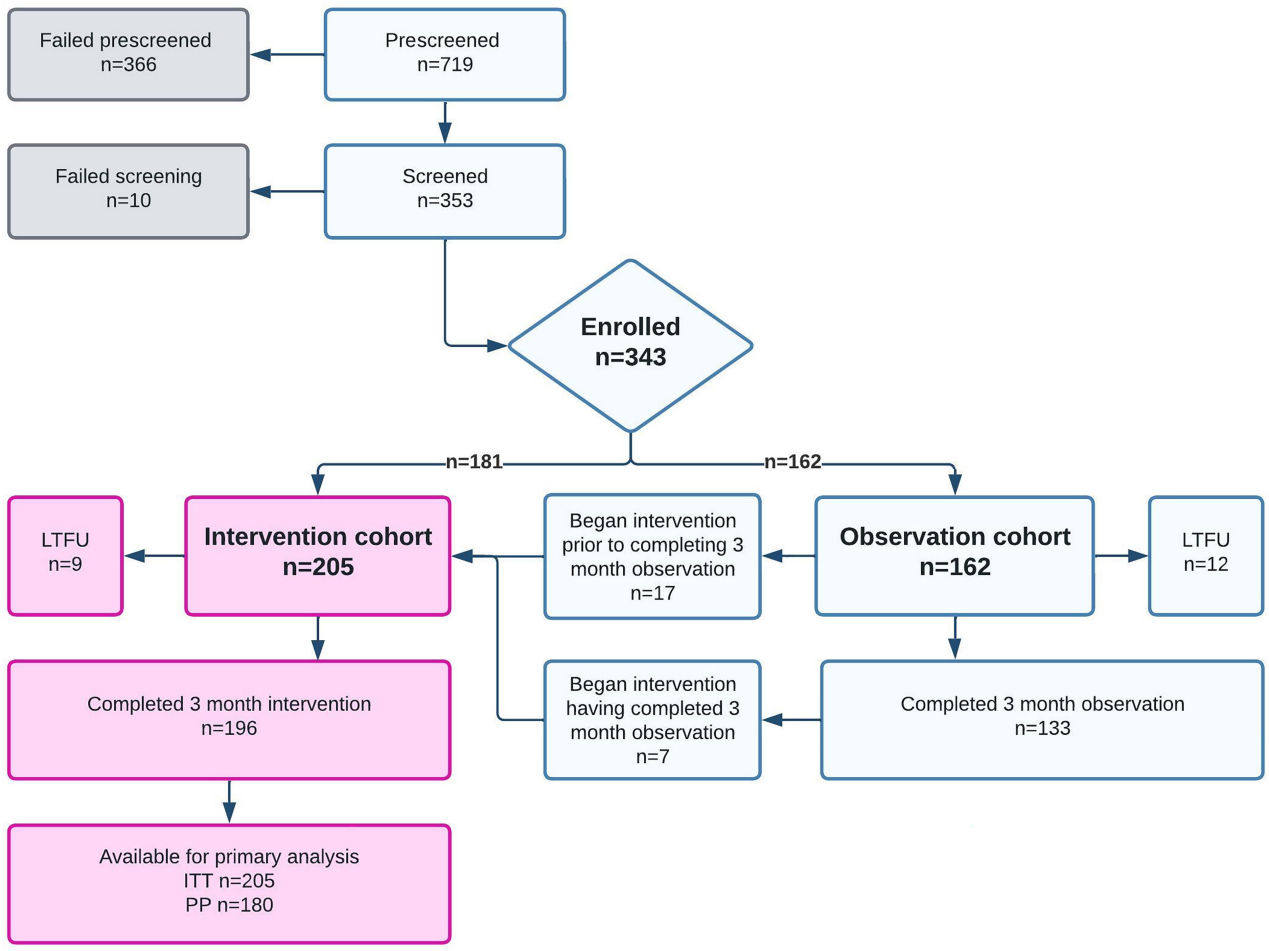
CONSORT flow diagram. CONSORT indicates ITT, intention to treat (analysis population); LTFU, loss to follow‐up; and PP, per protocol (analysis population).

Between October 2020 and July 2021, a total of 343 participants were screened. A summary of participant disposition is shown in Figure 2. A total of 162 participants with controlled BP at baseline were initially enrolled in the observation cohort. A total of 205 participants were enrolled into the intervention cohort. Of these, 181 started with uncontrolled BP at baseline and a further 24 became eligible for the intervention cohort with uncontrolled BP, having initially been enrolled into the observation cohort. All participants were followed up over 3 months. November 10, 2021, was the last date of follow‐up for intervention participants and November 19, 2021, for observation participants.

The 2 cohorts of participants had similar characteristics, shown in Table 1, apart from their baseline BP values and a higher proportion of participants who were not treated for hypertension at baseline in the intervention cohort.

**Table 1:** Demographic and Baseline Characteristics

	Cohort	
	Intervention (N=205)	Observation (N=162)
Age, y, mean (SD)	60.1 (11.0)	61.6 (9.2)
Sex		
Female, n (%)	68 (33.2%)	57 (35.2%)
Male, n (%)	137 (66.8%)	105 (64.8%)
Race		
Asian, n (%)	25 (12.2%)	14 (8.6%)
Black, n (%)	20 (9.8%)	19 (11.7%)
Mixed, n (%)	1 (0.5%)	2 (1.2%)
Other^a^, n (%)	8 (3.9%)	6 (3.7%)
White, n (%)	149 (72.7%)	120 (74.1%)
Unknown/ not reported, n (%)	2 (1.0%)	1 (0.6%)
BMI (kg/m^2^)†, mean (SD)	28.8 (5.3)	28.5 (4.9)
SBP (mm Hg)*, mean (SD)	141.9 (9.7)	126.0 (7.3)
DBP (mm Hg)*, mean (SD)	87.0 (8.1)	77.7 (6.4)
HR (bpm)*, mean (SD)	71.5 (10.9)	69.4 (9.5)
Current smoking		
Nonsmoker, n (%)	124 (60.5%)	104 (64.2%)
Previous smoker, n (%)	59 (28.8%)	44 (27.2%)
Current smoker, n (%)	21 (10.2%)	13 (8.0%)
Unknown/not reported, n (%)	1 (0.5%)	1 (0.6%)
Diabetes, n (%)	19 (9.3%)	21 (13.0%)
Kidney dysfunction, n (%)	1 (0.5%)	1 (0.6%)
Peripheral arterial/vascular disease, n (%)	3 (1.5%)	0
Hypercholesterolemia, n (%)	42 (20.5%)	45 (27.8%)
Previous stroke, n (%)	0	1 (0.6%)
Previous myocardial infarction, n (%)	3 (1.5%)	0
Previous percutaneous coronary intervention, n (%)	0	1 (0.6%)
On amlodipine (<10 mg) at baseline, n (%)	62 (30.2%)	74 (45.7%)
Class of hypertensive medication at baseline		
Alpha blocker, n (%)	8 (3.9%)	4 (2.5%)
Angiotensin‐converting enzyme inhibitor, n (%)	66 (32.2%)	59 (36.4%)
Angiotensin receptor blocker, n (%)	46 (22.4%)	49 (30.2%)
Beta blocker, n (%)	16 (7.8%)	13 (8.0%)
Calcium channel blocker‡, n (%)	72 (35.1%)	80 (49.4%)
Diuretic, n (%)	26 (12.7%)	21 (13.0%)
Mineralocorticoid receptor antagonists, n (%)	2 (1.0%)	0
Number of classes of medication participants are taking at baseline		
None, n (%)	49 (23.9%)	10 (6.2%)
1, n (%)	86 (42.0%)	91 (56.2%)
2+, n (%)	69 (33.7%)	61 (37.7%)

BMI indicates body mass index; BP, blood pressure; DBP, diastolic blood pressure; HR, heart rate; CI, and SBP, systolic blood pressure.

* Baseline BP and HR were calculated as the mean of 7 consecutive days of home BP measurements leading up to and including the day preceding the baseline date for each participant.

^†^ Data are missing for BMI in 7 intervention participants and in 9 observation participants.

^‡^ Concomitant (nonamlodipine) calcium channel blocker use at stable dose occurred for 7 participants in the trial. Four participants were on felodipine, 1 on diltiazem, 1 on nifedipine, and 1 on lacidipine, all of whom continued at stable dose throughout the trial. Additionally, 3 participants switched felodipine for amlodipine equivalent at baseline, 2 who had been taking 5 mg felodipine switched to 5 mg amlodipine, and 1 who had been taking 2.5 mg felodipine switched to 2 mg amlodipine.

^a^ Participants selecting “Other” were given a free‐text field to provide additional information. Responses included Hispanic/Brazilian/Portuguese, Filipino, broadly European, Jamaican, Ethiopian (1), Egyptian, African/Caribbean.

The most commonly reported comorbidities of interest were hypercholesterolemia (20% of intervention cohort) followed by diabetes (9%). Antihypertensive concomitant medication use (other than amlodipine) occurred among 45% (92) of the intervention cohort, with the following distribution for number of antihypertensive drugs: 1, 29% (60), 2, 15% (31), 3, 1% (2), 4, 0.5% (1). Antihypertensive drug use at baseline by drug class is shown in Table 1.

Of the 205 intervention cohort participants, all were included in the main intention‐to‐treat primary end point analysis and safety analysis, and 180 were included in the per protocol population. One hundred ninety‐six intervention cohort participants completed the trial and 9 were lost to follow‐up, and a further 7 participants completed the trial with no BP measurements recorded in the past 7 days before the EOT. In some instances, participants had an additional titration teleconsultation (unscheduled) after the last scheduled titration at Week 12. This was done if the clinician deemed it appropriate for a patient to have an additional dose change in order to end the trial on a dose of amlodipine that was considered optimal for the posttrial dose continuation strategy. There were 23 participants (11.7% of the 196 who completed the intervention cohort) who had an additional dose adjustment at an unscheduled visit after the last scheduled titration visit at Week 12. Secondary end points were assessed on all participants with available data for each end point.

### Trial Intervention

Adherence to trial interventions was high, with 84% (164/196) of participants adherent to BP self‐monitoring routines and 94% (185/196) of participants adherent to medication usage (self‐reported). Adherence to BP self‐monitoring was 75% (70/93) for those under 60 and was 91% (94/103) for those 60 years and older, and adherence to amlodipine medication was 94% (87/93) for those under 60 and 95% (98/103) for those 60 years and older. A post hoc assessment across age groups showed a similar level of adherence to BP self‐monitoring across ages up until 60 years (75% had 80% or higher, 70/93) and thereafter a substantial increase in adherence was apparent, with 89% (50/56) of those aged 60 to 69 having >80% adherence and 96% (46/48) of those aged 70 years and older having >80% adherence. A high level of adherence to the completion of routines in‐app was sustained over the course of the trial (Table 2). Those who finished the trial with controlled BP had slightly higher adherence to BP monitoring compared with those not controlled at EOT, 88% compared with 83%, respectively.

**Table 2:** Percentage of Intervention Cohort Participants with 80% or Higher Adherence to BP Data Entry Between Each Trial Visit, by Subgroups of BP Control at End of Treatment

	Before Week 1	Week 1 to Week 2	Week 2 to Week 4	Week 4 to Week 6	Week 6 to Week 8	Week 8 to Week 10	Week 10 to Week 12	Week 12 to EOT	Overall
All (n=189)*	88.4%	93.0%	90.0%	89.4%	90.0%	84.1%	81.0%	79.9%	83.7%
No BP control EOT (n=87)	86.2%	89.7%	87.4%	85.1%	87.4%	80.5%	79.3%	80.5%	82.8%
BP control EOT (n=102)	90.2%	96.1%	92.2%	93.1%	92.2%	87.3%	82.4%	79.4%	88.2%

BP indicates blood pressure; and EOT, end of treatment.

* The total number in this table is 189 intervention cohort participants who completed the trial and had available BP measurements at the EOT. A participant was classed as adherent to BP data entry for a particular routine if they entered at least 1 BP measurement. Each day consisted of 2 routines: 1 in the morning and 1 in the evening.

Overall patient retention in the intervention cohort was 96% (196/205 completing treatment). Adherence to BP self‐monitoring in the observation cohort over the 3‐month duration was 76% (101/133).

### Outcomes

#### Primary and Main Secondary Outcome

**Figure 3:**
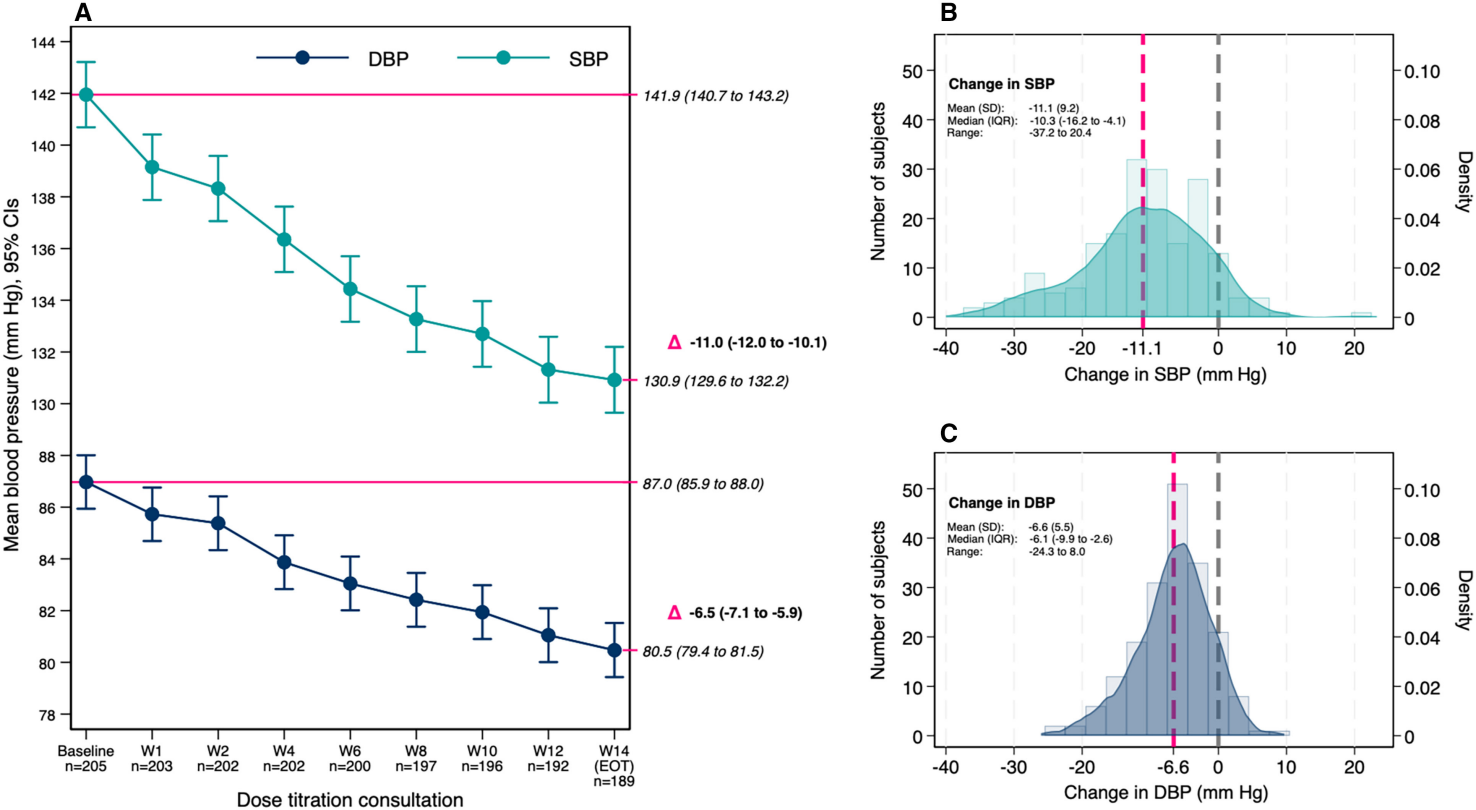
Primary and main secondary outcome—blood pressure at each scheduled remote consultation and change in blood pressure between baseline and the end of the trial (Week 14) in the intervention cohort. **A**, Estimated mean BP and mean change from linear mixed model with random participant component. **B**, Distribution of SBP change from baseline and the end of the trial (Week 14). **C**, Distribution of DBP change from baseline and the end of the trial (Week 14). BP indicates blood pressure; DBP, diastolic blood pressure; EOT, end of treatment; IQR, interquartile range; SBP, systolic blood pressure; and W, week.

Mean BP in the intervention cohort fell from 142/87 (SBP/DBP) to 131/81 mm Hg (SBP reduced by 11 [95% CI 10–12] and DBP reduced by 7 [95% CI, 6–7] mm Hg, *P*<0.001 for both) in the intention‐to‐treat population. The time course and distribution of BP changes from baseline to EOT are shown in Figure 3. Estimated BP changes were consistent when repeating the analysis on the per protocol population consisting of 180 participants who had at least 24 BP measurements available within 7 days of EOT, BP fell from 141/87 (SBP/DBP) to 130/80 mm Hg (SBP reduced by 11 [95% CI, 10–12] and DBP reduced by 7 [95% CI, 6–7] mm Hg, *P*<0.001 for both). Similarly, repeating the primary analysis on the subcohort of participants who were not already on amlodipine at baseline and who did not have a history of intolerance to amlodipine (n=123) made no difference in the patterns of BP reduction, falling from 143/87 (SBP/DBP) to 131/80 mm Hg (SBP reduced by 12 [95% CI, 10–13] and DBP reduced by 7 [95% CI, 6–8] mm Hg, *P*<0.001 for both).

**Figure 4:**
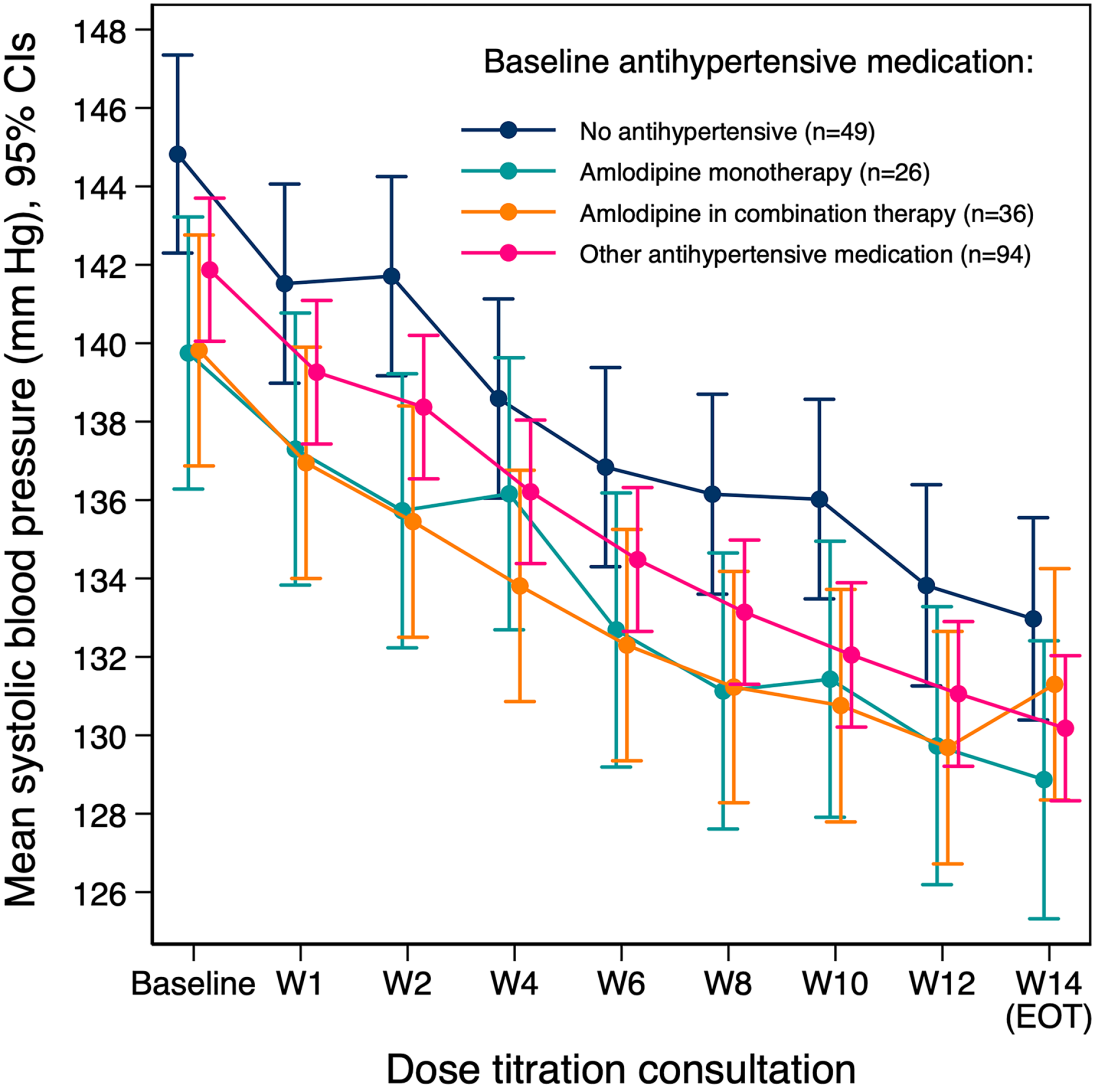
Mean change in systolic blood pressure by subgroups of participants grouped by their baseline antihypertensive medication. EOT indicates end of treatment; and W, week.

In a subgroup analysis of participants on different antihypertensive medication at baseline in the intervention cohort, although baseline SBP was slightly different between groups, the mean estimated reduction in SBP was very similar in trend and in magnitude over the course of the trial (Figure 4). In all subgroup analyses undertaken, there was no evidence for differing changes in SBP between groups.

#### Secondary and Other Outcomes

**Figure 5:**
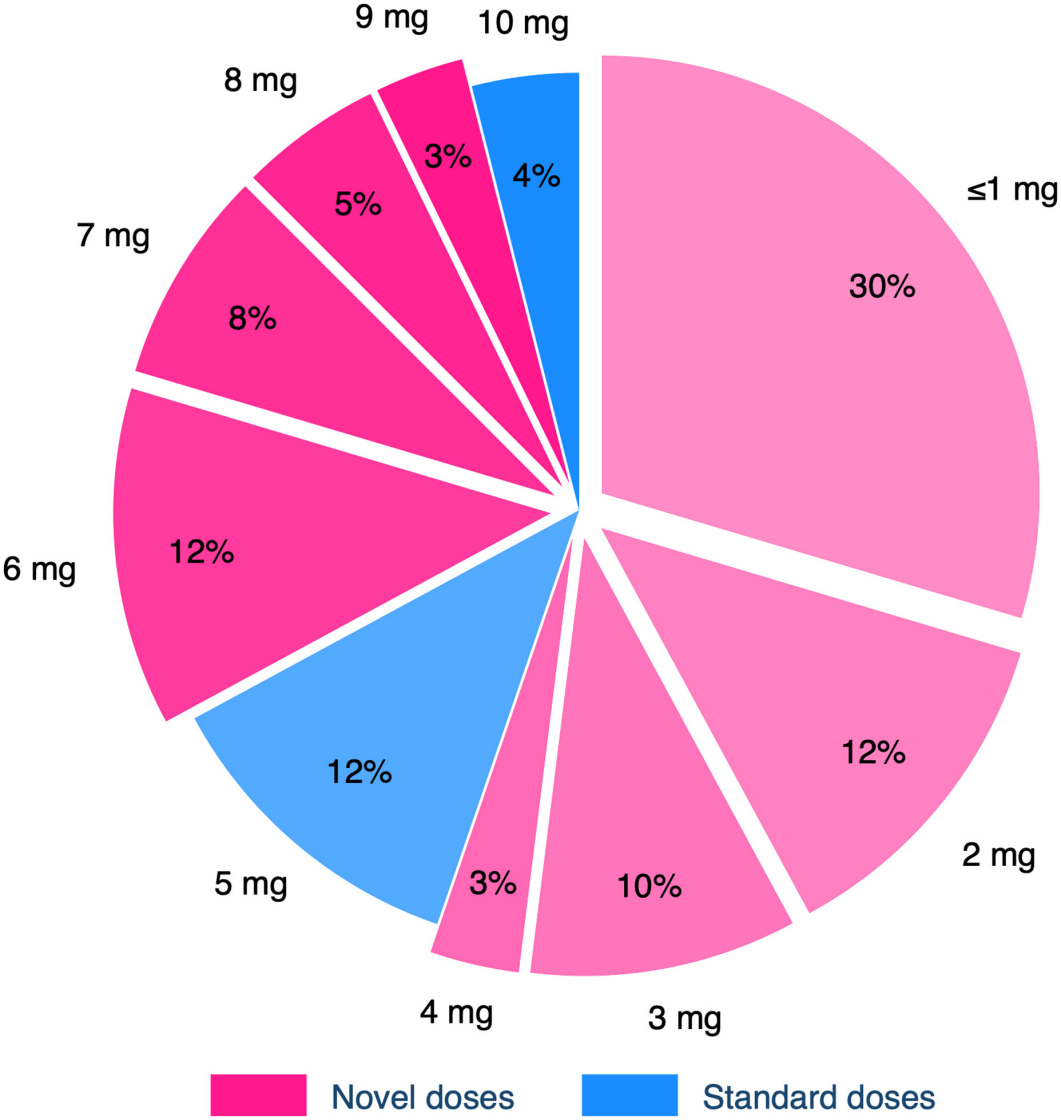
Distribution of novel and standard amlodipine doses that participants were prescribed when they first achieved blood pressure control. The figure includes 152 participants who achieved blood pressure control during the trial. The “≤1 mg” group contains some fractional doses: 2 participants on 0.5 mg and 1 participant on 0.25 mg.

**Figure 6:**
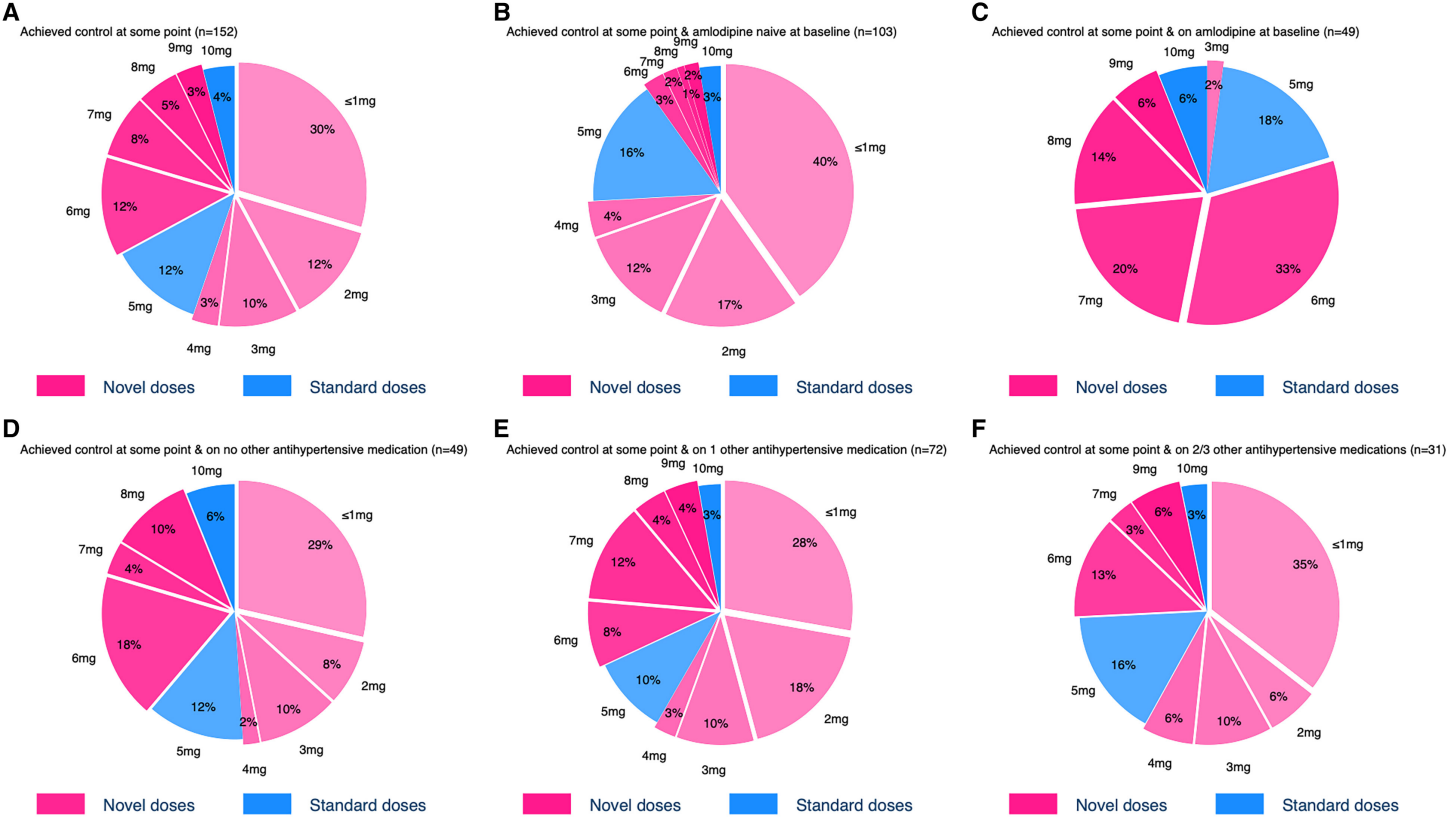
Distribution of novel and standard amlodipine doses that participants were prescribed when they first achieved blood pressure control for subgroups by baseline amlodipine use and by number of background antihypertensive medications received during the trial. The “≤1 mg” group contains some fractional doses: 2 participants on 0.5 mg and 1 participant on 0.25 mg. **A**, Achieved control at some point (n=152). **B**, Achieved control at some point & amlodipine naive at baseline (n=103). **C**, Achieved control at some point & on amlodipine at baseline (n=49). **D**, Achieved control at some point & on no other antihypertensive medication (n=49). **E**, Achieved control at some point & on 1 other antihypertensive medication (n=72). **F**, Achieved control at some point and on 2/3 antihypertensive medications (n=31).

Most participants in the intervention cohort (152/189, 80%) achieved BP control (SBP <135 mm Hg and DBP <85 mm Hg) during the trial, and a high proportion (128/152, 84%) were on novel doses when first achieving control. Of these 128 participants, 35% (45) were controlled by 1 mg daily (Figure 5). The distribution of amlodipine doses participants were receiving when they first achieved control was similar irrespective of the number (0–3) of background antihypertensive medications that participants were receiving during the trial (Figure 6). Also, the incremental (mg) amount of amlodipine irrespective of whether they were receiving amlodipine at baseline or not.

The majority (113/128, 88%) of participants controlled on novel doses had experienced no peripheral edema before achieving BP control.

The median time to achieving BP target (either SBP <135 mm Hg and DBP <85 mm Hg, or drop in SBP ≥10 mm Hg, OR drop in DBP ≥5 mm Hg) was 16 days (95% CI, 13–21).

At EOT, BP control was attained by 54% (102/189) and BP target by 76% (143/189); at Week 8 corresponding outcomes were 47% (90/197) and 68% (134/197), respectively.

In the observation cohort, mean BP remained similar between baseline and 3‐month follow‐up, 125/77 (SBP/DBP) and 124/76 mm Hg, respectively, in those who completed 3 months follow‐up. When including the 17 participants who crossed over into the intervention cohort before completing follow‐up in the observation cohort, mean BP was again similar between baseline and the last trial visit attended while in the observation cohort, 126/78 (SBP/DBP) and 125/77 mm Hg, respectively.

Dose‐limiting side effects were experienced by 59% (112/189) of participants, with 23% (44/189) requiring dose reduction and 10% (19/189) requiring pausing of amlodipine dosing at various time points during the trial (5 temporarily and 14 being on 0 mg at EOT). At EOT, 45% (85/189) had achieved BP control without experiencing a dose‐reducing side effect.

There were 22 participants in the intervention cohort who had previous intolerance to amlodipine (18 were not on amlodipine at baseline, 4 were on 5 mg at baseline). The majority (16 out of the 18) of participants who had a history of amlodipine intolerance and who were not receiving amlodipine at baseline ended the trial on amlodipine: 1 on 1 mg; 2 on 2 mg; 4 on 3 mg; 1 on 4 mg; 3 on 5 mg; 1 on 8 mg; 1 on 9 mg; 3 on 10 mg at completion.

**Figure 7:**
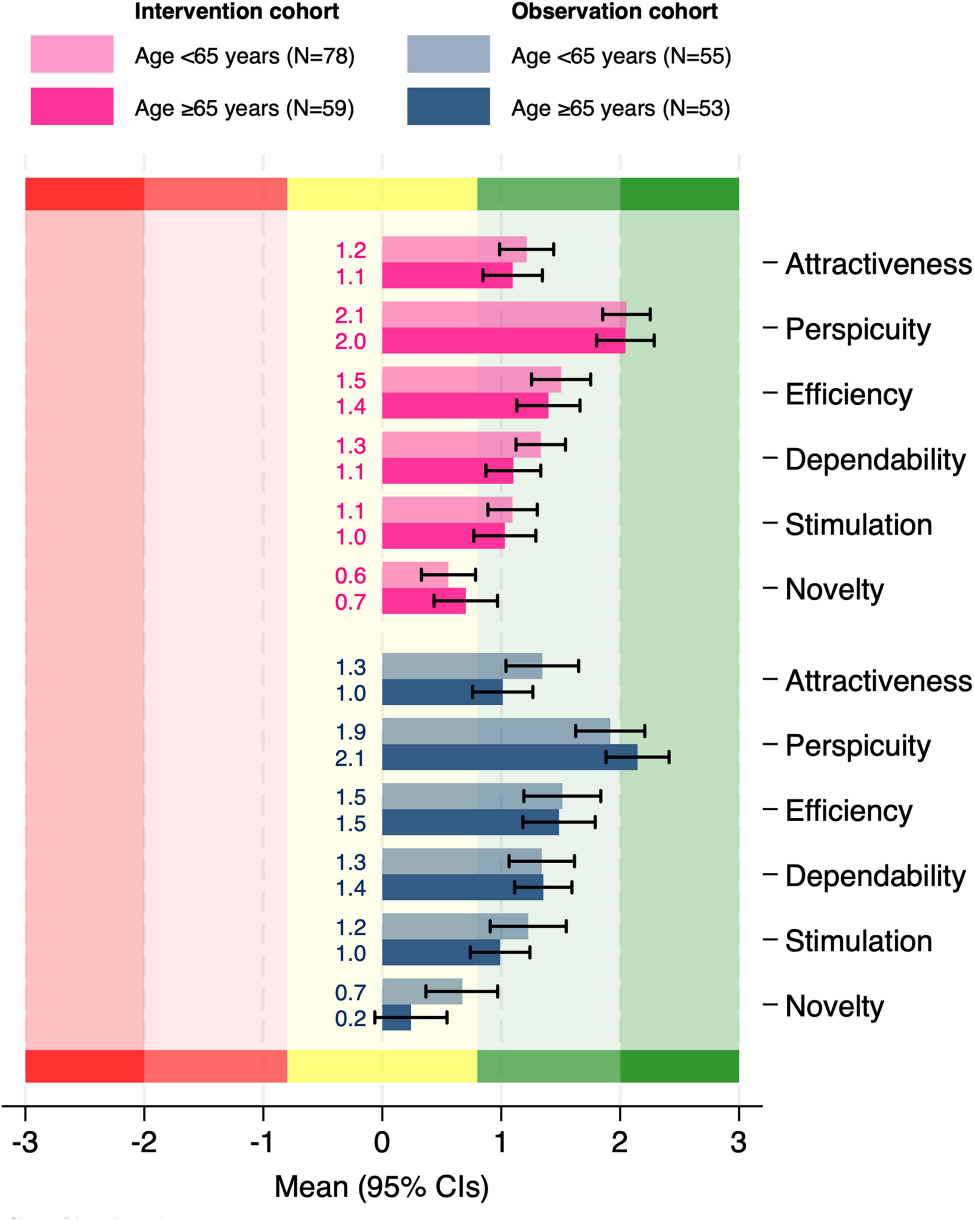
User Experience Questionnaire.

In the intervention cohort, the User Experience Questionnaire showed positive mean values for all scales and was consistent across age cohorts, with very positive evaluation (mean scores>1) for attractiveness, efficiency, dependability, and stimulation, with perspicuity (easy/understandable to use) scoring exceptionally highly (mean score>2) (Figure 7). Participants in the observation cohort reported very similar magnitudes of User Experience Questionnaire scores to the intervention cohort on average, also consistent across age cohorts.

### Adverse Events

There were 377 treatment‐emergent AEs in the intervention cohort, with severity distribution: 269 mild (71%); 101 moderate (27%); and 7 severe (2%) in intensity. The most common treatment‐emergent AEs by frequency were tiredness, fatigue, and weakness (n=76 events), gastrointestinal disorder (69), and headache (55) (Table 3). There were 5 serious AEs in the intervention cohort all considered unlikely or not related to trial drug (n=1 ST‐segment–elevation myocardial infarction, n=2 unstable angina, n=1 malignant breast tumor, n=1 nephrotic syndrome secondary to Waldenstrom's macroglobulinemia). There were no serious AEs, serious unexpected adverse reactions, or deaths. There were no trial withdrawals due to treatment‐emergent AEs. There was 1 serious AE (malignant melanoma) in the observation cohort.

**Table 3:** Treatment Emergent Adverse Events in the Intervention Cohort

Adverse event type	Number of participants (N=205)	Number of adverse events
Tiredness, fatigue, and weakness	70 (34%)	76
Headaches	52 (25%)	55
Ankle edema and other swelling	51 (25%)	52
Gastrointestinal disorder	45 (22%)	69
Skin reaction, rash, itching, and sweating	21 (10%)	23
Musculoskeletal and connective tissue disorder	16 (8%)	18
Dizziness	15 (7%)	15
Breathlessness and cough	13 (6%)	13
Anxiety and sleep disorders	11 (5%)	11
Blurred or abnormal vision and dry eyes	8 (4%)	8
Cardiac disorder	7 (3%)	7
Urinary frequency and nocturia	6 (3%)	7
General disorder	6 (3%)	6
Nervous system disorder	4 (2%)	5
Metabolism and nutrition disorder	4 (2%)	4
Erectile dysfunction	2 (1%)	2
Facial flushing	2 (1%)	2
Hearing impairment (tinnitus)	1 (<0.5%)	2
Hypotension	1 (<0.5%)	1
Labyrinthitis	1 (<0.5%)	1
Total	137 (67%)	377

## Discussion

This trial has demonstrated the ability to adjust and personalize drug dose to optimize clinical outcome from information recorded by participants in a dedicated smartphone app and transmitted securely to a clinician. Clinically significant reductions in BP were rapidly achieved from the first week using this novel titration protocol. Most participants' BPs were successfully controlled despite the cautious dosing regime based around 1 to 2 mg increments of amlodipine. Personalization of amlodipine dose improved BP control in participants, including those previously intolerant of conventional doses of amlodipine. This trial also established remote consent, enrollment, and assessment of baseline BP, based on twice‐daily measurements (in triplicate) over a 5 to 7‐day period with successful remote triaging of participants to intervention or observation based on control of hypertension. A minimum of 5 days and 24 BP measurements were required to calculate baseline BP and to determine eligibility for intervention or observation cohort. This is consistent with recently published data showing that 4.5 days of home monitoring is the minimum required for a reliable estimate of BP level.^20^

This trial has important strengths. It was the first trial to evaluate personalized novel amlodipine dose titration for mild–moderate hypertension under a remote medical management protocol. The trial tested the efficacy and safety of precision dosing of amlodipine incorporating novel doses up to the maximum licensed dose for hypertension. The trial included remote recruitment and triage into cohorts according to BP control, including an observation cohort. A limitation of the trial was that it did not include a control for comparison to the intervention cohort. Use of a placebo control was considered unethical, and usual care as a comparator during COVID‐19 lockdowns and restrictions was unpredictable, therefore a randomized controlled design was not feasible. Participants were required to possess a smartphone, which could limit the generalizability of the population and had the potential to lead to enrollment of a more affluent, younger, and educated population. However, smartphone ownership is high across demographic and economic groups.^21^

Most participants achieved BP control with novel doses of amlodipine. Small doses (from 1 mg) and small increments (1–2 mg) proved effective for gaining BP control. This effectiveness of small doses of amlodipine is consistent with a previous meta‐analysis^11^ and may reflect the sensitivity of veins to low doses of amlodipine, shifting blood volume from the high pressure (arterial) compartment into the venous system.^12^, ^22^, ^23^

It is important to note that although some participants continued to receive antihypertensive drugs during the trial (as per protocol), these were continued prescriptions from before the start of the trial and remained unchanged. Study drug/amlodipine was introduced as monotherapy or in addition to other BP‐lowering drugs that the participant may have been receiving. The only in‐trial change was dose titration of investigational medicinal product, amlodipine. An investigation was undertaken to assess whether those participants on additional hypertensive medications differed in achieving BP control compared with those on no other antihypertensive medication. The number of other antihypertensive medications made little difference to the distribution of amlodipine doses participants were on when they first achieved control. In addition, reductions in SBP were similar between participants taking other background antihypertensive medications compared with those on amlodipine alone. This supports the thinking that amlodipine may have an additive effect when added to other background medication.^11^

In their meta‐analysis of 345 randomized trials, Law and colleagues noted that “reduction in BP was only about 20% less at half standard dose of calcium channel blockers (predominantly amlodipine 2.5 mg) than at standard dose (5 mg), but adverse effects were much less common.”^11^ In this trial, the personalized novel dosing approach enabled effective management of emergent side effects including peripheral edema, the leading cause of patient discontinuation with amlodipine, to the extent that no trial discontinuations occurred that were associated with side effects. Patients with hypertension commonly experience unwanted treatment effects and being able to minimize or avoid side effects through precision dosing may improve long‐term adherence to treatment. In the very different setting of patients in secondary care with multiple drug intolerances, a case series was published using an atypical protocol that incorporated some fractional and liquid medications including nifedipine with examples of patient benefit.^24^

This trial's optimized 1 to 10 mg precision dosing regimen delivered efficacy (47% with BP control and 68% BP responders at 8 weeks) within the mid to upper range of outcomes reported in the literature for standard of care: 5 to 10 mg amlodipine (28%–54.9% BP control and 21.1%–73% BP responders).^6^, ^25^, ^26^, ^27^, ^28^, ^29^ Precision dosing delivered incremental improvement through to the end of the titration period (54% controlled and 76% responders at Week 14). In standard of care early dose titration studies, 61% to 88% of patients required titration from 5 to 10 mg amlodipine due to lack of BP control.^30^, ^31^, ^32^ Of the participants who achieved control by EOT, 48% of these were controlled on lower doses (1–5 mg) illustrating the potential of determining the optimal dose on an individual patient basis, informed through aggregated daily BP self‐monitoring and side effect reporting.

It is acknowledged that the absence of a comparator group means it is not possible to measure the amount of BP response attributable to amlodipine intervention directly, that is distinct from other factors including regression to the mean, placebo effect, and changes in lifestyle/behavior and other factors that may influence BP. However, the magnitude in BP change observed over the trial duration is encouraging, as it is far greater than other studies have observed in a placebo group.^28^, ^29^ Moreover, the minimization of regression to the mean was sought through the trial design. The trial benefited from using a high number of BP measurements to calculate a baseline BP for trial inclusion that was more likely to reflect individuals' true underlying BP levels, that is, reducing measurement error, reducing the likelihood of recruitment of participants based on random high BP measurements, and hence reducing the likely effect of regression to the mean from this cause.^33^

In the minority of patients not successfully controlled by amlodipine dose titration, whether as antihypertensive monotherapy or in addition to other BP‐lowering drugs that the participant may have been receiving, the addition of another antihypertensive drug may be warranted according to the clinician's judgment. Indeed some patients may require treatment with 2 or more drugs to achieve BP control and some treatment guidelines now recommend initial combination therapy. However, where the combination includes a calcium channel blocker such as amlodipine, managing tolerability and unwanted effects through optimal dose provision can present a significant obstacle, which this dose titration approach may address.

Although the TASMIN H4 trial showed a small, statistically significant benefit from self‐monitoring compared with usual care at 13 months, the addition of telemonitoring gave no clear additional benefit.^34^ Similarly, BP HOME, a large, recent randomized trial showed that standard self‐measurement of BP was not improved by using a BP measurement device paired with a smartphone application.^35^ In the United Kingdom, the Home BP study added a preplanned series of drug uptitrations for those patients in intervention, as well as alerts for clinicians if BP warranted uptitration, and achieved worthwhile reductions in BP related to increased titration of medication in the intervention group.^36^ An individual patient‐level meta‐analysis of self‐monitored BP interventions has already shown that the highest level individual support (cointerventions) provided to participants is linked to greater BP reductions.^7^ The intervention in PERSONAL‐CovidBP would be in their highest category (level 4).

This optimized precision dosing regimen delivered 100% retention rate to amlodipine treatment. No participants discontinued from the trial due to treatment‐related side effects over the 14‐week trial period. For standard of care, several studies have reported the percentage (0%–4%) of participants receiving amlodipine who discontinued during the treatment period due to side effects at 8 weeks of treatment.^37^, ^38^, ^39^, ^40^, ^41^, ^42^ Two studies examining 12 weeks of treatment (6% discontinued) and 2 studies focusing on 26 weeks of treatment (15%–33%) have also reported rates of discontinuation due to side effects increasing over time.^10^, ^43^, ^44^, ^45^ In this trial, adherence over the 14‐week trial period was high (94%), whereas the scientific literature is lacking in studies reporting adherence to amlodipine monotherapy. Adherence to antihypertensive medications has been widely assessed, with a number of high‐quality systematic reviews and meta‐analyses assessing adherence through the use of validated questionnaires and prescribing records, reporting adherence rates: between 51% and 64%, far lower than seen in this trial.^46^, ^47^, ^48^, ^49^ Encouragingly the adherence rate of older participants was actually higher than their younger peers, which is fortunate as the cardiovascular events in outcome trials using amlodipine are dominated by those over 65 years of age.^50^

A small number of studies have assessed adherence to recording of BP by telemonitoring. These studies show adherence rates of 40% to 72% among patients reporting BP more than 80% of the time with telemonitoring in periods ranging from 3 to 20 weeks.^51^, ^52^, ^53^, ^54^ Adherence in the trial was high, with 84% of participants adherent to BP self‐monitoring routines captured in the smartphone app record over the 14‐week trial period. Adherence to BP recordings in the observation cohort was also higher than reported in previous studies and not related to age.

As may be apparent, these are very high adherence levels in both groups, and overall app use was very well maintained. Adherence to the app remained high over the course of the trial, reducing slightly over time. In the Intervention cohort, the proportion of participants with 80% or higher BP data entry (at each routine) was 88% in the first week of the trial, remaining high at around 90% until there was a slight decline after Week 8 to 80% in the final 2 weeks of the trial.

There is an important question as to whether higher levels of BP measuring and engagement lead to better BP outcomes for patients, again adherence to BP data entry was high in general, but there was a slight difference in adherence between those who achieved BP control at EOT and those who did not. For example, in those achieving BP control by EOT, the proportion of participants with 80% or higher BP data entry (at each routine) was 93% in the first 2 weeks and 79% in the past 2 weeks, and in those who did not achieve BP control at EOT, the proportion was 88% in the first 2 weeks and 80% in the past 2 weeks. Therefore, adherence to reporting BP and amlodipine dosage appears to be similar whether participants achieved BP control or not.

Higher levels of AEs were observed than in previously published studies given the required daily self‐reporting of side effects by participants. Results from this trial agree with the daily angina scoring on an electronic diary recently developed for the ongoing ORBITA‐2 (A Placebo‐Controlled Trial of Percutaneous Coronary Intervention for the Relief of Stable Angina) trial in stable angina and suggest an underrepresentation of AEs in standard of care studies and the need to improve the design of future studies to ensure the capture of AEs for standard of care.^55^, ^56^ In standard of care trials, AEs have been typically reported at clinic visits at 4‐ and 8‐week time points. In the trial AEs were assessed at least twice weekly and side effects were entered daily in the smartphone app that in turn informed AE reporting. With standard of care there are participant discontinuations due to AEs, whereas in this trial there were no such discontinuations. Moreover, being able to adjust dose with precision to minimize drug intolerance enables participants to remain on therapy rather than discontinue.

The recent Food and Drug Administration precision dosing public meeting highlighted the potential importance of developing more precise dosing for drug‐disease targets where the potential outcome from under‐ or overdosing could result in serious morbidity.^57^ A senior Food and Drug Administration physician indicated this could be the third major milestone (the age of dosing individualization) in drug development and regulation after the ages of safety in 1938 and efficacy in 1962. Feedback‐based precision dosing for amlodipine and other drugs offers the ultimate dosing solution. In this trial, the treatment benefit appears to be associated with a high level of adherence, minimizing treatment discontinuation, leading to lowered BP and the prospect of long‐term control and cardiovascular event prevention. The psychological impact of abandoning a drug because it was intolerable, rather than trying to optimize its use, may reduce patients' convincement about treatment for this asymptomatic condition. More precise amlodipine dosing as trialed offers the prospect of improved patient outcomes and reduction in major cardiovascular events (stroke, myocardial infarction, death).

## Conclusions

In conclusion, the ability to define dose response at individual patient level for both BP control and side effects allows precision control of BP while minimizing or avoiding side effects. Precision dosing provides a means to improve a drug's risk–benefit profile in real world use. The data generated in this trial can be used to develop a product that will deliver precision control of BP at population health scale. Precision dose titration enabled by a dedicated smartphone app offers significant benefits enabling patients to personalize and optimize their therapy routine. Personalized digital plus drug combination health care solutions may offer the prospect to improve outcomes for patients and clinicians with significant potential utility for hypertension and other indications.

## Sources of Funding

The trial was funded by Innovate UK (project number: 105319‐23487) and Closed Loop Medicine.

## Disclosures

Authors M. Taylor, L. Richardson, J. Siddle, G. Timlin, and P. Goldsmith are employed by and receive income from Closed Loop Medicine. N.R. Poulter has received financial support from several pharmaceutical companies that manufacture blood pressure‐lowering agents, for consultancy fees (Servier), research projects and staff (Servier, Pfizer) and for arranging and speaking at educational meetings (AstraZeneca, Lri Therapharma, Napi, Servier, Sanofi, Eva Pharma, Pfizer, Alkem Laboratories, and Glenmark Pharma). He holds no stocks and shares in any such companies. He was supported by the National Institute for Health Research Senior Investigator Awards, Biomedical Research Centre funding, and the British Heart Foundation Research Centre Excellence Award. Dr McManus has worked with Omron Healthcare and Sensyne on telemonitoring interventions for which his institution receives funds including licensing. He is a National Institute for Health Research Senior Investigator and receives support from Oxford and Thames Valley National Institute for Health Research Applied Research Collaboration.
